# Assessing the naturalness of a restored coal mine area on the Loess Plateau, China

**DOI:** 10.1371/journal.pone.0219447

**Published:** 2019-07-12

**Authors:** Hong Yao, Jianjun Ma, Yongjun Fan, Xiuli Chen, Meirong Tian

**Affiliations:** 1 College of Life Science, Lang Fang Normal University, Lang fang, China; 2 Baotou Teacher’s college, Biological Science and Technology Institute, Baotou, Inner Mongolia, China; 3 Nanjing Institute of Environmental Sciences, Ministry of Ecology and Environment of China, Nanjing, China; CAS, CHINA

## Abstract

The Loess Plateau in China is an important area for mineral resources and therefore heavily exploited. As a measure to solve the conflict between conservation and development, ecological restoration has attracted more and more attention. More methods are needed to assess their effectiveness in achieving ecological and social goals. To adequately assess the effectiveness of natural restoration, the naturalness index (*NI*) has been developed to evaluate restoration effectiveness based on the Soil nutrient index (*SNI*), community composition index (*CCI*), and community succession index (C*SI*). By developing and applying of the *NI* to an open-pit mining area on Loess plateau, northwest China, the results show that: (i) In the study area, the cumulative dominance index of perennial grasses, the community function index, soil organic matter, and soil hydrolysable nitrogen greatly explained the community development. (ii) All the indicators values have changed with the increase of revolution time, the value of *SNI* increase obviously than the *CCI* and *CSI* comparing with the control plot, which indicated that the soil nutrient could be completely restored more easily. (iii) According to the Logistic Growth Model between *NI* and restoration time, it can be deduced that an ecosystem similar to the original ecosystem could be established after about 29 years of natural restoration.

## Introduction

The restoration of degraded ecosystems are currently receiving increased attention for which could Improving ecosystem health [1]. Ecological restoration includes reconstruction and evaluation, and evaluation predicts the effectiveness of our restoration work. The need for methods to evaluate effectiveness in achieving ecological and social goals increases, especially where restoration was an agricultural after use [2–3]. So ecological evaluation is particularly important. The conservation and restoration of degraded ecosystems were currently receiving increased attention [4]. There are many study aspects for ecological restoration evaluation, mainly in scale, object, method and index system. Having a macro-scale perspective, Lupp et al. (2013) studied the naturalness of forest landscape management and change [5]. Szilassi (2017), using natural assets as an indicator system, studied the naturalness of vegetation restoration at landscape scale in Hungary area [6]. From three scales of archipelago (Galapagos), island (El Hierro) and map sheet, Machado comprehensively elaborated on the construction of the index system of naturalness [7]. On a small scale, related studies consider factors such as soil engineering, vegetation disposition, species selection [8–10], methods and technologies of artificial restoration, and changes in species diversity in the process of vegetation recovery [11–13].

New advances have been made in the quantitative evaluation index system for degraded ecosystems [14–15], but the restoration process as such is still not included. Although in recent years, environmental impacts have increasingly been measured and monitored, with considerably progress in the used methodologies, these methodologies are only rarely combined with the restoration of ecosystems. In terms of research methodologies, researchers often only use a single index, such as species richness or species diversity, instead of considering the total species or the habitats. Such an approach impedes comprehensive measurements of space-time coupling effects on the species, community, and environment in the process of recovery [16], that is, the complete performance of a successful recovery. According to many scholars, the recovery of the structure and function is the ultimate goal of ecological restoration [17–20]. It is therefore necessary to establish an evaluation method that can assess not only the state of ecological restoration, but also reveals the historical and spatial characteristics.

The naturalness index (*NI*) of a community, which refers to the distance or similarity degree between the actual communities and their original state, is used to evaluate the specific community status, including the soil, the community composition, and the community succession [21]. At present, studies employing the *NI* mainly focus on forest ecosystems [22–28], while the evaluation of the naturalness of reclaimed land in coal mines is relatively less [29].

The types and patterns of a community depend on both natural changes and human activities within the ecosystem [30]. By considering the present and past changes within a given system, we can not only evaluate the ecological restoration status, but also clearly reveal the impacts of anthropogenic activities on these factors.

Based on this understanding, we assessed the plant communities of the Heidaigou open-pit coal mine reclamation land, located on the Loess Plateau region in China, using the ecological background as evaluation standard. To analyze the naturalness of the area, we employed fuzzy mathematics on parameters such as soil nutrition, community composition, and community succession.

## Materials and methods

### Study area

**Fig 1:**
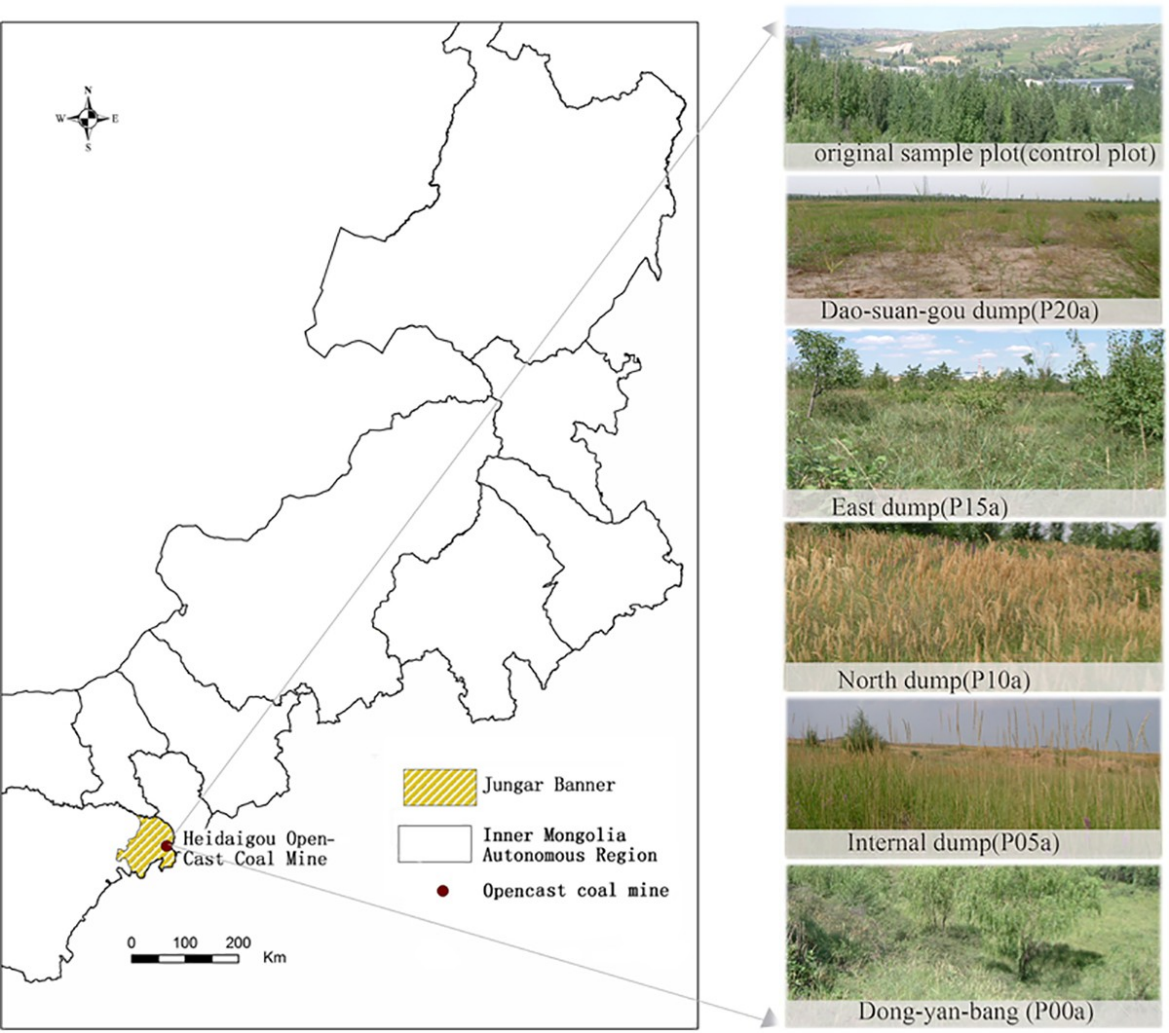
The sketches of sample plots, Jungar Banner, Inner Mongolia.

The study area was located in eastern Jungar Banner, Erdos City, Inner Mongolia Autonomous Region, Northwest China (111°13'- 111°20′ E, 39°43'- 39°49′N) (Fig 1). The climate is warm temperate, semi-arid continental climate. Average annual temperature is 7.2°C, with an average annual precipitation of 231–460 mm.

The zonal vegetation of the hilly slopes is characterized as warm temperate steppe vegetation. *Stipa bungeana* grassland is the original vegetation type, but only occurs in the form of several small fragments. The remaining fragments are mainly composed of a *Stipa bungeana* + *Cleistogenes squarrosa* + forb community; the main species are *Stipa bungeana*, *Cleistogenes squarrosa*, *Leymus chinensis*, *Thymus mongolicus*, *Lespedeza davurica*, *Medicago ruthenica*, *Astragalus scaberrimus*, and *Oxytropis chiliophylla*. In addition, we identified *Heteropappus altaicus*, *Artemisia frigida*, and *Ixeris gracilias*. The landscape is monotonous, with a simple community composition.

This study was carried out in the field of restored Heidaigou coal mine area which was State-owned Land and did not involve endangered or protected species. Meanwhile, because this study supported by the Special Scientific Research Projects in Environmental Protection and Public Welfare Industry, so the government of Jungar Banner permitted and approved this study.

### Experimental design and vegetation sampling

Experiments were performed from July to September 2015. For this, five plots, representing five different restoration starting time points, were established, located in the following areas of the former coal mine: Dao-suan-gou dump (*P*_*20a*_), East dump (*P*_*15a*_), North dump (*P*_*10a*_), Internal dump (*P*_*05a*_), and Dong-yan-bang dump (*P*_*00a*_). For comparison purposes, an undisturbed plot within the mining area was selected as a control plot (Table 1).

**Table 1:** Basic properties of the sample plots.

Monitoring points	Location		Altitude (m)	Description of sample land
	E	N		
Original sample plot (*Control plot*)	111°17′05″	39°47′22″	1259	Not severely interfered with by humans. Perennial grasses included *Stipa krylovii*, *Leymus secalinus*, and *Cleistogenes squarrosa*.
Dao-suan-gou dump (*P*_*20a*_)	111°15′45″	39°47′60″	1266	Formed in 1995, natural reclamation for 20 years. Perennial grasses included *Stipa bungeana*, *Leymus chinensis*, *Cleistogenes squarrosa* and *Agropyron cristatum*.
East dump (*P*_*15a*_)	111°17′52″	39°46′44″	1263	Formed in 2000, natural restoration for 15 years. Perennial grasses were *Stipa bungeana*, *Leymus chinensis*, *Cleistogenes squarrosa* and *Agropyron cristatum*.
North dump (*P*_*10a*_)	111°16′33″	39°47′57″	1277	Formed in 2005, natural reclamation for 10 years. Perennial grasses include *Calamagrostis epigeios*, *Leymus secalinus*, *Cleistogenes squarros and Poa sphondylodes*.
Internal dump (*P*_*05a*_)	111°16′19″	39°47′18″	1250	Formed in 2010, natural reclamation for 5 years. Perennial grasses: *Calamagrostis epigeios*.
Dong-yan-bang dump (*P*_*00a*_)	111°16′18″	39°46′57″	1268	Formed in 2015, natural reclamation for 0 year. Including of annual herb.

Note: *P*_*00a*_ indicates the plot with a natural restoration time of 0 years, the others are the same.

All plots met the following requirements: i) in the restored plots, restored soil thickness was similar, with similar surface soil properties. The slope was less than 5 degrees, and all plots were subjected to similar impacts of groundwater; ii) the control plot showed a topography similar to that of the restored plots; it was not subjected to any anthropogenic activities.

#### Community composition investigation

For vegetation surveys, we established three transects in each plot, each transect was 100 meters, with a distance of 5 m between transects. In each transect, eight quadrats (1 m × 1 m) were established, and each quadrat had an interval of 10 meters. We assessed the following parameters: total coverage, community biomass and coverage, species density, species height, and species biomass.

#### Vegetation succession and community development

The dominant species of the community were assessed to determine the succession stages and the natural succession degree, which were then used to analyze the dynamic characteristics of the community.

#### Soil investigation

In each plot, 10–15 samples points were established in an "S" shape, and soil samples were taken at a depth of 0–30 cm. Based on the findings of previous studies [22][24], we used the parameters community composition, community succession, and soil properties as indices to evaluate the success of ecological restoration.

### Community composition index (CCI)

#### Cumulative dominance (SDH4)

The cumulative dominance (*SDH*_4_) can reflect the functional status and distribution pattern of different species within a community. In grassland, for example, perennial grasses play an important role in the community composition and function. Based on this, we used the cumulative dominance (see Eq 1) of the perennial grasses to illustrate the development of the community:

$$SDH_{4}=(C^{\prime }+H^{\prime }+D^{\prime }+W^{\prime })/4$$ (1)

Where *SDH*_4_ is the cumulative dominance of the perennial grass; *C*′,*H*′,*D*′ and *W*′ are relative coverage, relative height, relative density, and relative biomass (dry weight), respectively. The values of *C*′,*H*′,*D*′ and *W*′ are determined as follows:

Relative coverage (*C*′) = Total coverage of perennial grass∕Total coverage;

Relative height (*H*′) = Total height of perennial grass/Total height;

Relative density (*D*′) = Total density of perennial grass∕Total density;

Relative biomass (*W*′) = Total biomass of perennial grass∕Total biomass.

#### Richness index (Pa)

The Patrick richness index (*Pa*), which does not consider the number of individuals, i.e., ignoring the effects on community diversity, refers to the number of species in a quadrat [31]; it can be calculated as follows:

Pa=S (2)

Where S is the number of species.

#### Shannon-Wiener diversity index (H)

The Shannon-Wiener diversity index is calculated according to the following equation:

$$H=-\sum_{i=1}^{m}P_{i}lnP_{i}\;(i=1,2,3,\ldots n)$$ (3)

Where *P*_*i*_ = *N*_*i*_/*N*, *N* is the total number of individuals of all species and *N*_*i*_ is the number of individuals of species *i* (this does also apply to the following equation).

#### Pielou evenness index (JP)

The Pielou evenness index (*JP*) refers to the distribution of different species within a community; it therefore reflects the evenness of the species composition and is expressed as follows:

$$JP=-(\sum_{i=1}^{m}P_{i}lnP_{i})/lnS$$ (4)

### Community succession index (CSI)

#### Community similarity index—Jaccard index

Based on the presence or absence of certain species within the communities, to compare the similarities between the different communities, the communities that experienced less interference by humans were chosen as the basis and compared with the other communities. The Jaccard index is calculated as follows:

$$C_{j}=c/(a+b-c)$$ (5)

Where *C*_*j*_ is the Jaccard index; c is the number of common species of two communities, a and b are the number of their own species of two communities.

#### Community function index

The functional characteristics of both dominance and coverage can reflect the degree of community succession. The dominance and coverage of the control community, which is in a certain succession stage, are relatively stable. Therefore, the community function index (see formula 6) can be used as an important index to partition the community succession series [32]:

$$D_{j}=\sum_{i=1}^{m}\left(I_{i}\times D_{i}\right)\times \frac{V}{M}$$ (6)

where *D*_*j*_ is the community function index; *I*_*i*_ is the life cycle of species i, determined according to the life form, namely, an annual plant scores 1, a biennial plant scores 2, chamaephytes, hemicryptophytes, and cryptophytes score 10, shrubs and small trees (3–5 m) score 50, large trees (> 5 m) score 100; Di is the dominance index; M is the number of species; V is the total coverage of the community. If total coverage is 80 or 100%, V = 0.8 or 1, respectively.

Different life-form functional groups play different roles in vegetation succession, and perennial herbs significantly contribute to the stability (including structure, function, and productivity) of a community. Using a scale of 1–9 (the specific method is described below in 2.6.2), the weights of all life forms were determined, with values of 0.4545, 0.2727, 0.1818, and 0.0909. The *I*_i_ values were modified by weight, using Eq 7.

$$I_{i}^{\prime }=I_{i}\times L_{j}$$ (7)

Where $I_{i}^{\prime }$ is the modified life cycle of species *i*; *L*_*j*_ is the weight of life form. The modified equation reads as follows:

$$D_{j}^{\prime }=\sum_{i=1}^{m}\left(I_{i}\times L_{j}\times D_{i}\right)\times \frac{V}{M}$$ (8)

Where $D_{j}^{\prime }$ is the modified community function index.

### Soil nutrient index (SNI)

Soil nutrient indices refer to the physical and chemical properties that directly affect plant growth and community succession, including soil pH, available phosphorus, hydrolysable nitrogen, available potassium, and organic matter.

### Naturalness index (NI)

Analytic hierarchy processes were used to construct the *NI* [33].

#### Sensitivity analysis of evaluation index

The coefficient of variation (*CV*) was used to analyze the sensitivity of the selected 11 indices [34]. Based on the sensitivity, the final indices were determined.

#### Determining the weight of the evaluating indices

Currently, evaluation of the environmental quality is typically performed using the Analytic Hierarchy Process (*AHP*), and based on previous studies, the reliability and the scientific nature of the AHP are satisfactory. In this study, the *AHP* was used to apportion the weight of the indicators, and the ratio scale method was used to construct the judgment matrix.

To determine the maximum characteristic value and the characteristic vector required to obtain the relative weight of each factor, the random consistency ratio (*CR*) of the judgment matrix was calculated. When the *CR* is less than 0.1, the judgment matrix is satisfactory; in particular, the distribution of weight is reasonable and is calculated as the square root of the product of all the elements in the judgment matrices, according to the following equation:

$${\bar{W}}_{i}=\sqrt[{n}]{\prod_{i=1}^{n}a_{ij}}\;(i=1,2,3,\ldots n)$$ (9)

$$W_{i}=\frac{\bar{W_{i}}}{\sum_{i=1}^{n}\bar{W_{i}}}\;(i=1,2,3,\ldots n)$$ (10)

Where *W*_*i*_ is the approximate value of the proper vector, which also represents the relative weights of indices.

#### Original data standardization

Because the dimensions of the indices are not uniform, the factors must be standardized to overcome parameter incomparability, using the following standardization formula:

$$S_{i}=(x_{i}-x_{min})/(x_{max}-x_{min})$$ (11)

Where *S*_*i*_ is the standard value of the evaluation index; *x*_*i*_ is the measured value; *x*_*max*_ is the maximum measured value; and *x*_*min*_ is the minimum measured value.

#### Comprehensive evaluation of the NI

According to the standard value and to the weight and grade scores of the indices, we can obtain the naturalness index using the following equation, with results being randomly distributed in the range of 0–1:

$$NI=\sum_{c=1}^{m}f_{c}w_{c}+\sum_{s=1}^{n}f_{s}w_{s}+\sum_{t=1}^{p}f_{t}w_{t}$$ (12)

Where *NI* is the naturalness index; *f*_*c*_, *f*_*s*_ and *f*_*t*_ are the standard values of the community composition, community succession, and soil index, respectively; *w*_*c*_, *w*_*s*_ and *w*_*t*_ are weights of *f*_*c*_, *f*_*s*_ and *f*_*t*_ respectively; and *m*, *n*, and *p* are the numbers of the corresponding indices.

### Screening and determining each index

The sensitivity grading of the 11 indices was as follows: Coefficient of variation (CV) ≤ 10%: not sensitive; 10% < CV < 30%: low sensitivity; 30% < CV < 60%: sensitive; CV > 60%: highly sensitive. According to the analysis results of the sensitivity, 9 indices were selected as the naturalness indices because of their sensitivity, namely the cumulative dominance index, the Patrick richness index, the Shannon-Wiener diversity index, the Pielou evenness index, the community similarity coefficient, the community function index, the content of organic matter, the content of available phosphorus, and the content of hydrolyzable nitrogen.

### Calculation of the weight of each index

The scale of 1–9 was applied to analyze the importance of the criterion layers (B) and index layers (C) and to obtain the weights of the criterion layers (B_1_-B_3_) and the index layers (C_1_-C_9_) (Table 2).

**Table 2:** Naturalness valuation index system and the weights for naturalness indexes.

Target layer (A)	Criteria layer (B)	Respective weight	Index layer (C)	Respective weight	Total weight
*NI* (A)	*CCI* (B_1_)	0.3	Cumulative dominance index (C_1_)	0.375	0.1125
			Patrick richness index (C_2_)	0.125	0.0375
			Shannon-Wiener diversity index (C_3_)	0.250	0.0750
			Pielou evenness index (C_4_)	0.250	0.0750
	*CSI* (B_2_)	0.2	Community similarity coefficient (C_5_)	0.333	0.0666
			Community function index (C_6_)	0.667	0.1334
	*SNI* (B_3_)	0.5	Content of organic matter (C_7_)	0.500	0.2500
			Content of available phosphorus (C_8_)	0.167	0.0835
			Content of hydrolysable nitrogen (C_9_)	0.333	0.1665

## Results

**Fig 2:**
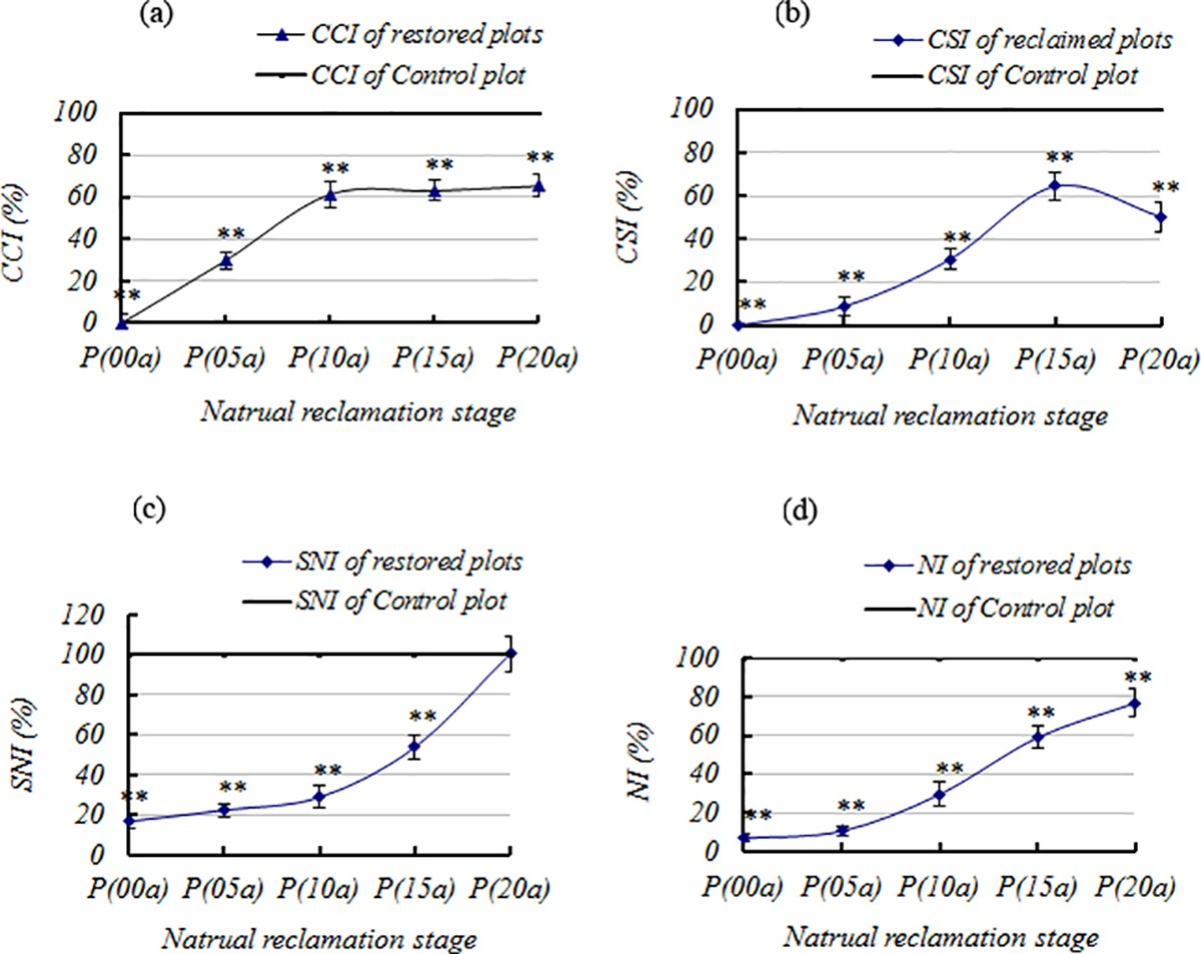
(a)—(d) The change of the *CCI*, *CSI*, *SNI* and *NI* in different natural stage respectively. Note: * indicates lack of sincerity to the control (*p* > 0.05); *indicates significant difference (*p* < 0.05); ** indicates highly significant difference (*p* < 0.01).

First, the real values were standardized. Second, according to the above formulas, the dynamic characteristics of *CCI*, *CSI*, *SNI*, and *NI* were revealed (Fig 2(A)–2(D)), with further details in the S1 Table.

As seen in Fig 2(A) and Fig 2(B), *CCI* and *CSI* showed consistent trends. With increasing revegetation time, both *CCI* and *CSI* increased. From 1995 to 2015, during the first 10 to 15 years of natural restoration, the *CCI* and *CSI* first increased and subsequently stabilized. While the *CCI* increased over a period of 10 years, the *CSI* increased over 15 years. The first growth stage was termed as the “lottery competition period”, with a large influx of species within a relatively short time and without dominant species. In the stable phase, the so-called “dominant competition period”, the community was characterized by the decrease in species and the appearance of dominant species; this phase lasts longer than the “lottery competition period”. Based on the “dominant competition period” as shown in Fig 2(A) and Fig 2(B), *CCI* and *CSI* had more complexity, and the *CCI* showed no significant differences (*p* > 0.05). In the 1995 plot, the *CSI* (50.3%) was significantly lower than in the 2010 plot (64.4%), possibly because of the increasing community complexity over time.

Although the plant communities had developed over a period of 20 years, the *CCI* and *CSI* values were significantly lower than the values of the control plot, reaching only about 60% compared to the control. However, over this period, the soil nutrient levels could be completely restored (Fig 2(C)). Compared with the recovery of the soil nutrient status, *CCI* and *CSI* had an obvious time lag, exhibiting the variation of “rising—stagnation—rising”. Due to the lack of a longer time sample, determining how long the “lag time” will continue, i.e., when the third phase should begin, warrants further study.

The rapid recovery of the soil properties could be explained as follows: i) soil nutrients are accumulated over time when anthropogenic activities are absent; ii) the background values of the studied soil are relatively low, with a total nitrogen content < 0.01% and an organic matter content < 1%), although the soils are rich in phosphorus and potassium. The recovery of the vegetation is therefore more complex than soil (N, P, and K) restoration.

With increasing restoration time, the *NI* gradually increased (Fig 2(D)). The control plot had the highest level (100%), followed by the 1995 plot (76.77%), 2000 plot (58.90%), 2005 plot (29.61%), 2010 plot (10.60%), and 2015 plot (6.95%).

**Fig 3:**
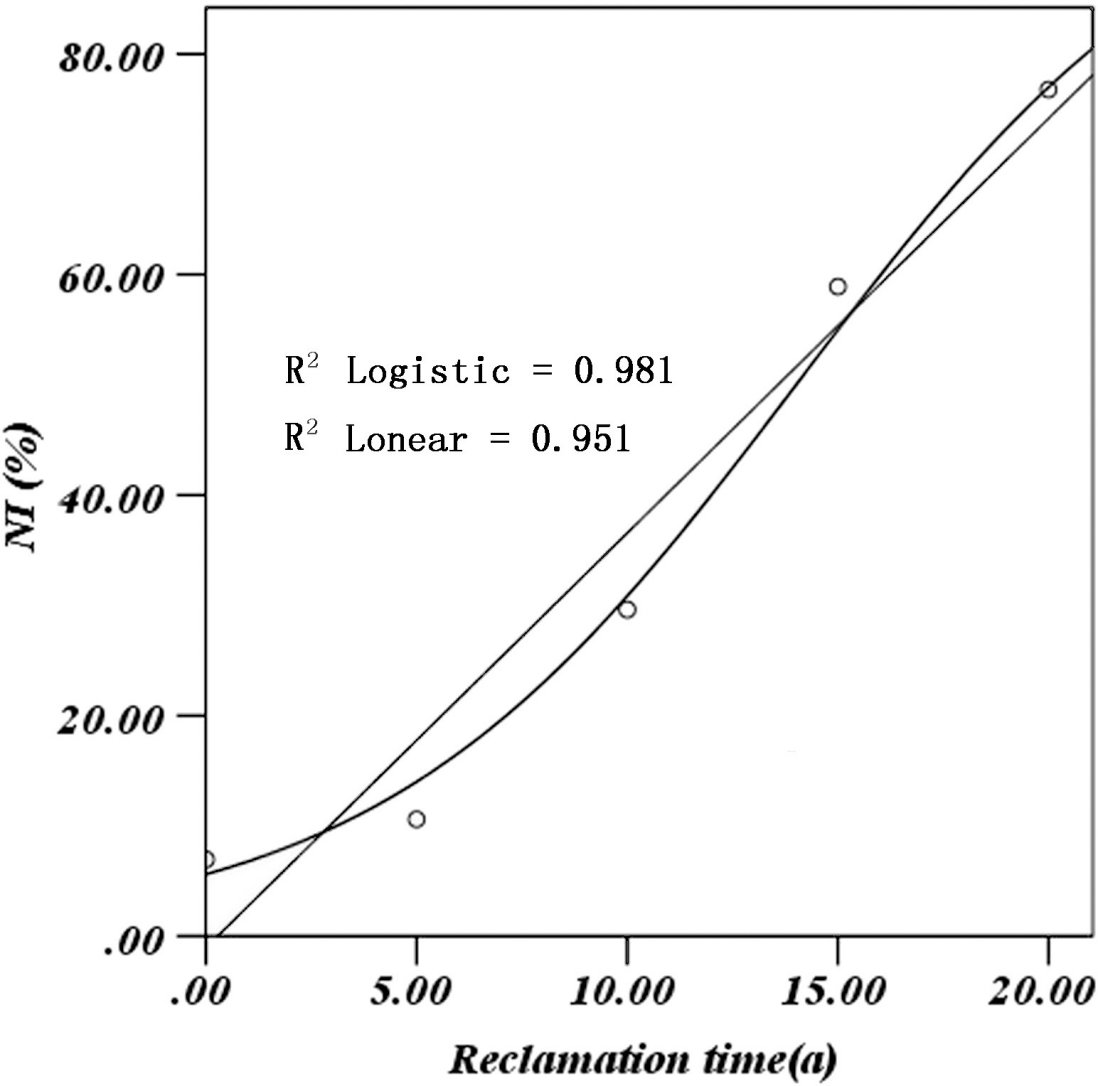
Fitting curve of naturalness index (*NI*) and reclamation time.

To predict the time required for the restored sites to reach a structure and similarity similar to those of the control site, in Fig 3, the x-axis was defined as reclamation time, while the y-axis was still *NI*. Regression analysis (Used IBM SPSS 20.0) was performed between restoration time and *NI* (Fig 3).

In Fig 3, the actual and the predicted values of Logistic growth model were highly correlated (R^2^ = 0.981); the integral formula of the Logistic growth model:

$$N=\frac{K}{1+e^{a-rt}}$$ (13)

Where *N* is the naturalness index; *t* is the restoration time; *r* is growth rate of the naturalness index; *K* is environmental capacity, in this article, that is the maximum value of naturalness index (100%); *a* is the constant; *e* is the constant, base of natural logarithm.

By regression analysis, the constant *a* and the growth rate *r* can be determined. Deforming and taking the logarithm on both sides of the Eq (13), according to the statistical method of a linear regression equation, *a* (2.8226) and *r* (0.2015) can be obtained.

The Logistic growth model was $NI(\%)=\frac{100\%}{1+e^{2.8226-0.2015t}}$. According to the formula, it was inferred that the ecosystem with functions and services similar to those of the original one can be achieved after natural restoration for about 29 years.

## Discussion

In this work we determined the *NI* of a community at different restoration stages, using various index systems and evaluation methods to evaluate ecological restoration of a former coal-mining site.

One difficulty in this approach is how to determine the evaluation criteria and the reference frame for ecological restoration [35], because desirable trajectory and outcome was often challenged by the unpredictability of ecological communities in the changing environment [36].

In this study, the “primary communities”, which were free or minimally influenced by anthropogenic activities, were used as reference communities. Based on the community composition, community succession, and soil conditions, we established a comprehensive evaluation system of *NI* of former coal mine sites at different recovery stages, including the cumulative dominance index, the Patrick richness index, the Shannon-Wiener index, the Pielou evenness index, the similarity index of community, the community succession index, organic matter, available phosphorus, and hydrolytic nitrogen. Ruiz-Jaén & Aide (2005) have argued that when evaluating ecological restoration, we must select at least two reference systems, and we must investigate attributes of diversity, community composition, and ecosystem processes clearly related to ecosystem function [37]. Therefore, in this study, each attribute was reflected by at least two variables.

Considering the history and the present conditions of all sites used in this study (restored and control sites), the background values, which are relatively stable, reflect the comprehensive identification of the coenotype and the community composition; therefore, the indicative function of the community background values is of a high practical significance for ecological restoration [38].

Yu et al. (2002) have proposed an index system consisting of the potential degree, restoration degree, and recovery rate, based on the composition, structure, and function of a given community [39]. The system is feasible both to evaluate the recovery of a degraded Karst community and to be used in long-term location studies. Li et al. (2007) have proposed the indicative index to assess the restoration of a degraded ecosystem [40]. Studies have suggested that the changes in the cumulative coverage were most obvious in each stage; therefore, this is the most indicative index, and the diversity index of tree species may also be used to indicate the progress of the degraded ecosystem.

Here, the study area (Loess Plateau) and the study object (warm temperate steppe community) differed from those used in previous studies [41–42]. On the Loess Plateau, the four evaluated indices, namely cumulative dominance index of perennial grasses, community function index, soil organic matter, and soil hydrolysable nitrogen, significantly contributed (over 66%) to the community development.

## Conclusions

The results of our study suggest that with increasing restoration time, the *NI* increases, which means that over time, the plant community more closely resembles the original one. Community composition, succession, and soil conditions adequately reflected the successional stage.

The evaluation index system of near-naturalness, constructed in this study, can objectively reflect the process of natural restoration; it can be divided into three categories: (i) community composition index (*CCI*), including cumulative dominance index, Patrick richness index, Shannon-Wiener index, Pielou evenness index; (ii) Community succession index (*CSI*), including community similarity coefficient, community succession degree; (iii) Soil nutrient index (*SNI*), including organic matter, available phosphorus, and hydrolyzed nitrogen.

In the course of natural restoration, the *CCI* and *CSI* went through two stages, an initial increase, followed by a stagnant phase.

We tried to develop a reasonable evaluation system of ecological restoration effects through small-scale experiments. As with any restoration program, in the study area, the goal was to obtain natural communities resembling, as far as possible, the original plant communities. In this sense, if ecosystem recovery is understood correctly, the outcome of restoration efforts can successfully be predicted, and research projects can be better designed. The measurement of ecosystem processes can provide important information about the recovery of a given ecosystem, enabling an estimation of the resilience of the ecosystem.

## Supporting information

S1 Table The value of *CCI*, *SNI*,*CSI* and *NI* at the different natrual reclamation stage. (XLS)
